# Mandibular Fracture Patterns at a Medical Center in Central Taiwan: A 3-Year Epidemiological Review

**DOI:** 10.1097/MD.0000000000009333

**Published:** 2017-12-22

**Authors:** Fu-Yu Lin, Chao-I Wu, Hsu-Tang Cheng

**Affiliations:** a Department of Neurology; b Division of Plastic and Reconstructive Surgery, Department of Surgery, China Medical University Hospital, China Medical University School of Medicine; c Division of Plastic and Reconstructive Surgery, Department of Surgery, Asia University Hospital, Asia University College of Medical and Health Science, Taichung, Taiwan.

**Keywords:** epidemiology, mandible fracture, Taiwan

## Abstract

Mandibular fractures constitute a major portion of maxillofacial trauma and may lead to considerable functional and aesthetic sequelae if treatment is inadequate or delayed. An epidemiology study on mandibular fractures may guide the preventive efforts of the Taiwan public health care system. Therefore, a retrospective review was conducted at a medical center in central Taiwan to evaluate the current mandibular fracture epidemiology.

The medical records and digitized radiographs of 198 patients who received treatment for mandibular fractures during a 3-year period (from October 2010 to September 2013) at a medical center in central Taiwan were reviewed to obtain demographic and injury data.

The average age was 29.4 years (3–82 years). Patients aged 21 to 30 years sustained the most mandibular fractures (62 patients, 31.3%). The overall sex distribution (male to female) ratio was 1.8. Motor-vehicle accidents (MVAs) were the most common mechanism of injury (162 patients, 82%), and scooter and motorcycle riders wearing partial-coverage helmets constituted the majority of patients. A chart review identified 198 patients with 335 mandibular fractures; 113 patients (57.1%) had multiple mandibular fractures. The most common fracture sites were the symphysis and parasymphysis regions (38.9%), followed by the condyle (26.0%), angle (14.3%), body (14.3%), and ramus (6.6%).

MVAs are the major cause of mandibular fractures in central Taiwan, and patients aged <30 years sustained the most mandibular fractures. Compared with previous studies, the present study has a higher percentage of women with mandibular fractures. In addition, inadequate mandibular protection by partial-coverage helmets may be a major reason for mandibular fractures most commonly localized in the symphysis and parasymphysis regions. The incidence and causes of mandibular fractures may reflect the trauma patterns within the community, thus facilitating the development of a preventive strategy for the socioeconomic and environmental background of central Taiwan.

## 1 Introduction

Mandibular fractures constitute a major portion of maxillofacial trauma because of its prominence, unique mobility, and location. Mandibular fractures, first described in 1650 BC in an Egyptian papyrus,^[1]^ may result in various functional and aesthetic sequelae, including malocclusion, poor mastication, chronic pain, and temporomandibular joint syndromes, if treatment is inadequate or delayed. Injuries associated with mandibular fractures range from minor face and head lacerations to lethal closed-brain injuries.^[2,3]^

The etiology and incidence of mandibular fractures vary with the different geographic regions, socioeconomic status, cultures, traffic rules, and study eras. Motor-vehicle accidents (MVAs) have been reported as the major cause of mandibular fractures in developing countries, and interpersonal violence has become the most common cause in many developed countries.^[4]^ However, information on the mandibular fracture patterns in Taiwan is limited.^[5]^

A mandibular fracture epidemiology study may facilitate the preventive work of the Taiwan public health care system.^[6]^ China Medical University Hospital and the affiliated level-I trauma center are located in downtown Taichung City in central Taiwan, where motorcycles and scooters are the main modes of public transport. We conducted a retrospective review with data from China Medical University Hospital to evaluate the current epidemiology of mandibular fractures.

## 2 Materials and methods

A retrospective review of all mandibular fractures reported at China Medical University Hospital over a 3-year period (from October 2010 to September 2013) was performed. Patients receiving treatment at the Department of Plastic and Reconstructive Surgery and the Department of Oral and Maxillofacial Surgery, the only 2 departments responsible for facial trauma care at the hospital, were included. This study was approved by the Institutional Review Board of China Medical University.

The medical records and digitized radiographs, including a panorex and/or bone algorithm computed tomography, were examined to obtain the relevant information. Patient data were recorded with respect to sex and age. The patients were categorized into 7 age groups: 0 to 10, 11 to 20, 21 to 30, 31 to 40, 41 to 50, 51 to 60, and >60 years. Injury etiology was recorded on the basis of the emergency room records and classified as MVA, fall, assault, and other causes. Accident details including mode of transport (bicycle, scooter, motorcycle, or car), presence or absence of a helmet, and helmet type (full coverage or partial coverage) were recorded for patients with an MVA history.

Fracture sites were assigned as 5 mandibular subunits, namely the condylar and subcondylar area, ramus, angle, body, and symphysis and parasymphysis, based on the documented radiographic and intraoperative findings. Data organization and analyses were performed using Microsoft Excel (Microsoft).

## 3 Results

From October 2010 to September 2013, a total of 198 patients with 335 mandibular fractures were treated at China Medical University Hospital.

### 3.1 Age and sex distribution

The average age was 29.4 years. The youngest and oldest patients were aged 3 and 82 years, respectively. Patients aged 21 to 30 years sustained the most mandibular fractures (62 patients, 31.3%) with a male to female ratio of 2.26. The 11 to 20 age group had similar numbers of patients (61 patients, 30.8%) with a male to female ratio of 1.44. The sex distribution of the study population over the 3-year period revealed that 127 (64%) men and 71 (36%) women experienced mandibular fractures, with an overall sex distribution (male to female) ratio of 1.8:1 (Table 1).

**Table 1:**
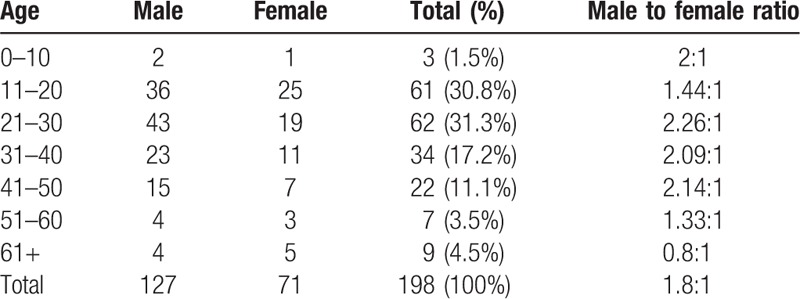
Mandibular fractures regarding age and sex.

### 3.2 Injury mechanisms

**Figure 1:**
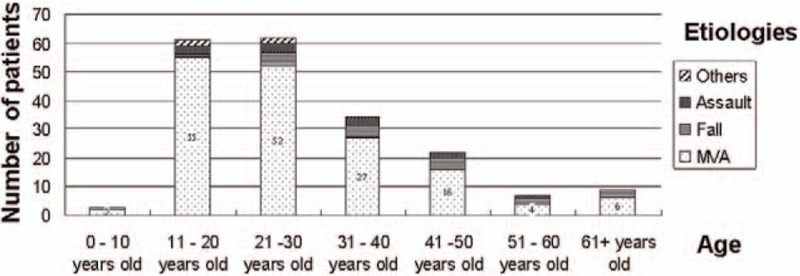
Mandibular fractures regarding age and etiologies. MVA = motor-vehicle accident.

**Figure 2:**
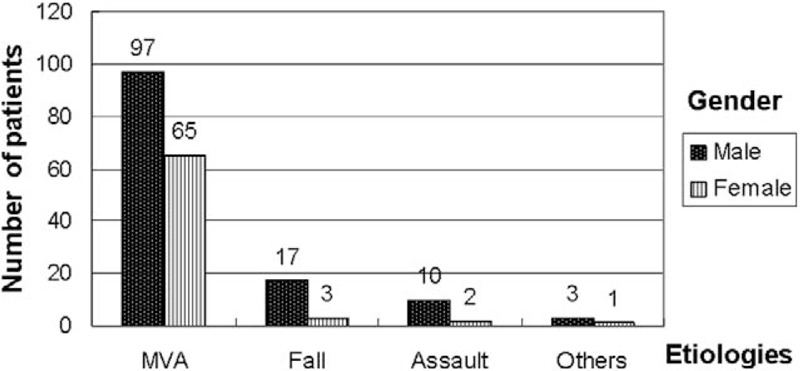
Mandibular fractures regarding sex and etiologies. MVA = motor-vehicle accident.

MVAs were the most common mechanism of injury (162 patients, 82%). In addition, MVAs were the most common cause of mandibular fractures in all age groups, predominantly in the 11 to 20 and 21 to 30 age groups (n = 55, 33.9% and n = 52, 32.0%, respectively; Fig. 1). MVAs were predominant in men (n = 97, 59.8%; Fig. 2). Fall and assault accounted for 10% (n = 20) and 6% (n = 12) of mandibular fractures, respectively. Among the remaining 4 cases (2%), 2 teenagers and 2 patients in the 21 to 30 age group experienced mandibular fractures due to sport-related and work-related injuries, respectively.

**Figure 3:**
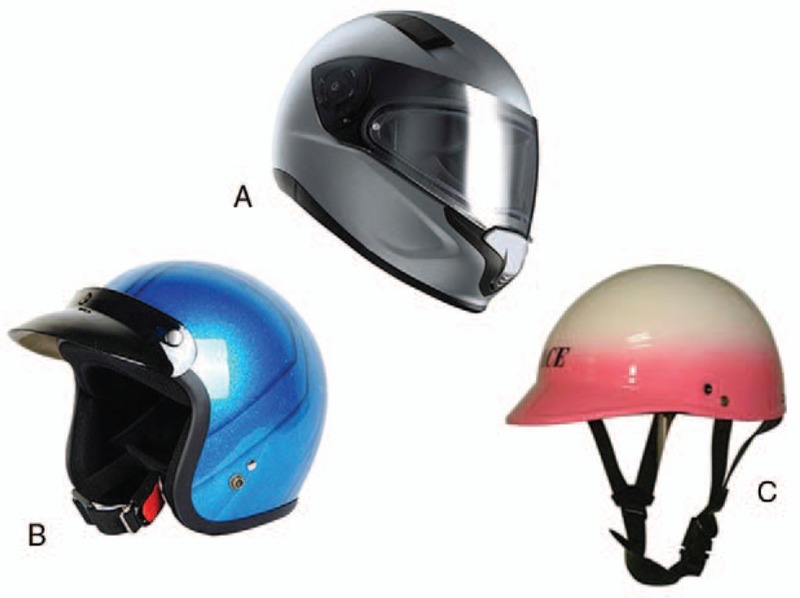
Common types of helmet worn among Taiwanese scooter/motorcycle riders: (A) full-covered helmet; (B) 3/4-covered helmet; (C) 1/3-covered helmet.

Among the 162 patients with a history of MVA-related mandibular fractures, 6 and 156 patients were bicycle and scooter/motorcycle riders, respectively; no car drivers had a history of MVA-related mandibular fractures. Of the 6 bicycle riders, only 2 patients had worn helmets, and none of the 156 scooter or motorcycle riders had worn full-coverage helmets (Fig. 3).

### 3.3 Fracture site and anatomy

**Figure 4:**
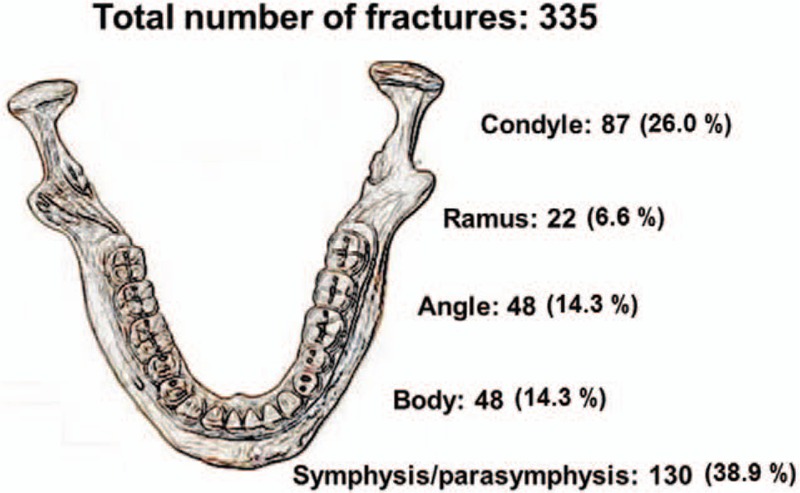
Distribution of fractures regarding anatomic location.

A total of 335 mandibular fractures were reported at the hospital, and 113 patients (57.1%) had multiple mandibular fractures. Of these 113 patients, 69 (61.1%) were men (Table 2). Symphyseal and parasymphyseal fractures were the most common (n = 130, 38.9%), followed by condylar (n = 87, 26.0%), angle (n = 48, 14.3%), body (n = 48, 14.3%), and ramus (n = 22, 6.6%) fractures (Fig. 4). Of the 130 patients with symphyseal and parasymphyseal fractures, 36 had single-site fractures, and others had combination fractures involving the condyle on 1 side (n = 24), the angle (n = 20), and condyles on both sides (n = 14).

**Table 2:**
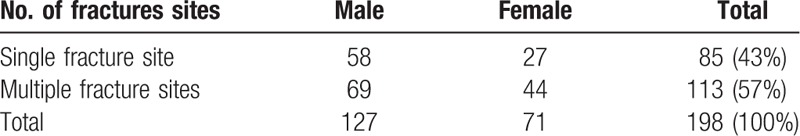
Sex and number of fractures.

## 4 Discussion

A total of 199 patients who presented with mandibular fractures from October 2010 to September 2013 at China Medical University Hospital were included in the present retrospective study. Although this study has some similarities with the previously reported large-scale studies in Taiwan and other countries,^[4]^ several present findings may highlight the unique influences of the geographic, cultural, and socioeconomic status of the study region.

In the present study, patients in the 21 to 30 age group (n = 62, 31%) had the most mandibular fractures. Yu et al^[5]^ evaluated facial fracture cases at Tri-Service General Hospital (Taiwan) between 1992 and 2000 and revealed that patients aged 16 to 30 years sustained approximately 60% of the study population's facial fractures. Similarly, most foreign studies on mandibular fracture epidemiology have reported that these fractures predominantly occur in the 21 to 30 age group.^[6–11]^ However, in the present study, the numbers of patients in the 11 to 20 and 21 to 30 age groups were almost equal (n = 61) with similar injury patterns. Although this may be due to the limited sample size, it emphasizes that safe traffic behaviors should be encouraged in adolescents and young adults.

In accordance with the previous studies on facial trauma, the present study showed a male predominance (127 male patients [64%]), with a sex distribution (male to female) ratio of 1.8:1. Notably, this study had a higher female incidence of mandibular fractures (36%) compared with those in Germany (25.6%),^[12]^ Canada (22%),^[6]^ Egypt (21%),^[10]^ Thailand (17%),^[7]^ and Turkey (16%; Table 3).^[4]^ Moreover, another study in Taiwan revealed that the sex distribution ratio (male to female) was 9:1 and 3:1 in 1982 and 1992 to 2000, respectively.^[5]^ Therefore, the incidence of mandibular fractures in females has increased in recent decades. This increase may be because motorcycles and scooters are the major modes of transport among the female citizens in Taichung City.

**Table 3:**
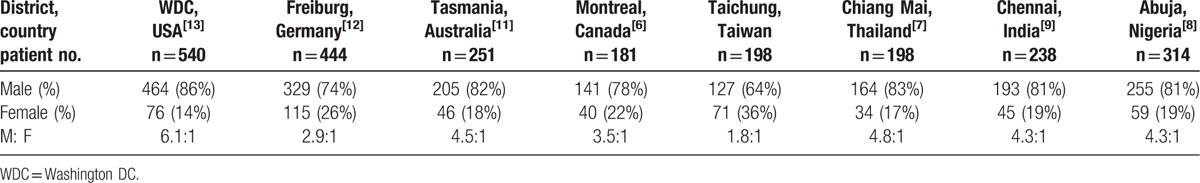
Sex distribution among different countries.

In addition, the popularity of partial-coverage helmets may be a reason for the high mandibular fracture incidence. Compared with full-coverage helmets, partial-coverage helmets significantly increased the risk of head injury in motorcycle riders.^[14]^ In addition to markedly reducing mid- and upper-face injuries, helmets may reduce the risk of head or brain injury by approximately two-thirds or more. However, helmets did not prevent lower-face injuries.^[15]^

MVAs have been reported as the major cause of mandibular fractures in developing countries, whereas interpersonal violence has become the most common cause of these fractures in many developed countries.^[4]^ In the present study, MVAs were the predominant (82%) mechanism of injury; other causes of mandibular fractures were relatively minor (Fig. 1, Table 4). Taiwan is still on the verge of becoming a developed country; the concerned authorities should focus on traffic regulations and public transportation system reforms.

**Table 4:**
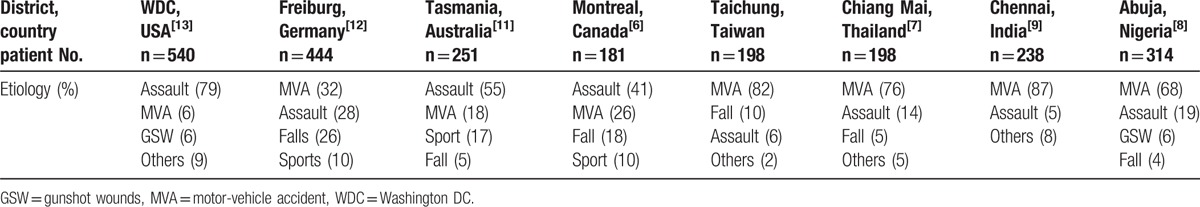
Etiology of mandibular fractures ranked according to frequency among different countries.

Fridrich et al^[16]^ revealed that motorcycle accident–related mandibular fractures are mainly observed in the symphysis and parasymphysis regions. Furthermore, the angle was the most common site of assault-related mandibular fractures. In our study, with the majority (82%) of the patients were MVA victims, symphysis and parasymphysis fractures were the most common (38.9%) sites of fracture. We present the fracture patterns in different districts and countries in Table 5.

**Table 5:**
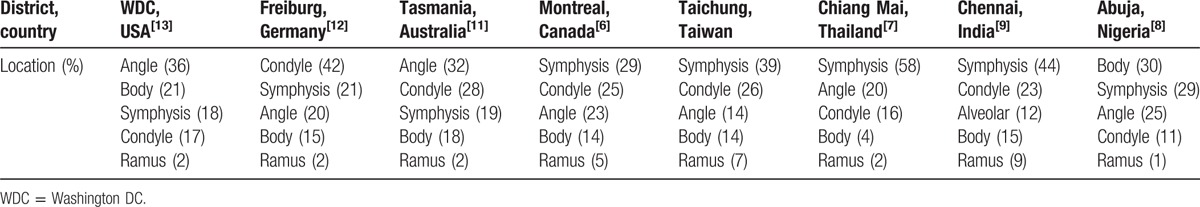
Location of mandibular fracture sites ranked according to number reported at each site among different countries.

Mandibular fractures potentially indicate serious injuries because high energy is required to disrupt the strong mandibular structure. Mandibular fractures may consume a substantial portion of the budget in the health care system compared with other injuries.^[17]^ Therefore, understanding the epidemiology of mandibular fractures will guide the preventive efforts required to reduce their incidence and resulting sequelae.

## 5 Conclusion

MVAs are the major cause of mandibular fractures in central Taiwan, and patients aged <30 years sustained the most mandibular fractures. Compared with previous studies, the present study has a higher percentage of women with mandibular fractures. In addition, inadequate mandibular protection by partial-coverage helmets may be a major reason for mandibular fractures most commonly localized in the symphysis and parasymphysis regions. The incidence and causes of mandibular fractures may reflect the trauma patterns within the community, and thus facilitating the development of a preventive strategy for the socioeconomic and environmental background of central Taiwan.
